# High pyrethroid-resistance intensity in *Culex quinquefasciatus* (Say) (Diptera: Culicidae) populations from Jigawa, North-West, Nigeria

**DOI:** 10.1371/journal.pntd.0010525

**Published:** 2022-06-21

**Authors:** Ahmed Idowu Omotayo, Musa Mustapha Dogara, Danjuma Sufi, Tasiu Shuaibu, Joshua Balogun, Salwa Dawaki, Bature Muktar, Kamoru Adeniyi, Nura Garba, Isah Namadi, Hafiz Abdullahi Adam, Shuaibu Adamu, Hamza Abdullahi, Abubakar Sulaiman, Adedayo Olatunbosun Oduola

**Affiliations:** 1 Molecular Entomology and Vector Control Research Laboratory, Department of Public Health and Epidemiology, Nigeria Institute of Medical Research, Yaba, Lagos, Nigeria; 2 Department of Zoology, Faculty of Life Sciences, University of Ilorin, Ilorin, Kwara State, Nigeria; 3 Department of Biological Sciences, Federal University Dutse, Dutse, Jigawa State, Nigeria; 4 Department of Biological Sciences, Federal University Kashere, Gombe State, Nigeria; Centers for Disease Control and Prevention, UNITED STATES

## Abstract

This study examined pyrethroid resistance intensity and mechanisms in *Culex quinquefasciatus* (Say) (Diptera: Culicidae) populations from Jigawa, North-West Nigeria. Resistance statuses to permethrin, lambda-cyhalothrin and alphacypermethrin were determined with both WHO and CDC resistance bioassays. Synergist assay was conducted by pre-exposing the populations to Piperonyl butoxide (PBO) using the WHO method. Resistance intensities to 2x, 5x and 10x of diagnostic concentrations were determined with the CDC bottle method. Species analysis and presence of knockdown mutation (Leu-Phe) were done using Polymerase Chain Reaction (PCR). Results showed that *Cx*. *quinquefasciatus* was the only *Culex* spp. present and “*Kdr*-west” mutation was not detected in all analyzed samples. Using WHO method, *Cx*. *quinquefasciatus* resistance to permethrin was detected in Dutse (12.2%) and Kafin-Hausa (77.78%). Lambda-cyhalothrin resistance was recorded only in Kafin-Hausa (83.95%) with resistance suspected in Ringim (90%). Resistance to alphacypermethrin was recorded in all locations. Pre-exposure to PBO led to 100% mortality to alphacypermethrin and lambda-cyhalothrin in Ringim while mortality to permethrin and alphacypermethrin in Dutse increased from 12.2% to 97.5% and 64.37% to 79.52% respectively. Using CDC bottle bioassay, resistance was also recorded in all populations and the result shows a significant positive correlation (R^2^ = 0.728, *p* = 0.026) with the result from the WHO bioassay. Results of resistance intensity revealed a very high level of resistance in Kafin-Hausa with susceptibility to lambda-cyhalothrin and alphacypermethrin not achieved at 10x of diagnostic doses. Resistance intensity was also high in Dutse with susceptibility to all insecticides not achieved at 5x of diagnostic doses. Widespread and high intensity of resistance in *Cx*. *quinquefasciatus* from North-West Nigeria is a major threat to the control of diseases transmitted by *Culex* and other mosquito species. It is a challenge that needs to be adequately addressed so as to prevent the failure of pyrethroid-based vector control tools.

## Author summary

Development of resistance to insecticide by mosquitoes has been identified to be a major challenge in the prevention and control of diseases transmitted by mosquitoes. This informs this study that investigated the level of resistance of *Culex* mosquitoes from Jigawa, North-West Nigeria to Pyrethroids. The main type of *Culex* mosquitoes found in the sampled area was *Cx*. *quinquefasciatus*. The *Cx*. *quinquefasciatus* populations were found to be resistant to permethrin, lambda-cyhalothrin and alphacypermethrin. Resistance in *Cx*. *quinquefasciatus* from the three LGAs is more pronounced to alphacypermethrin. The methods employed by the mosquitoes in developing resistance involve detoxification of the insecticides by metabolic enzymes. *Cx*. *quinquefasciatus* from the three LGAs were observed to be highly resistant and can withstand multiple of the recommended doses. This development whereby *Cx*. *quinquefasciatus* populations were highly resistant to these recommended insecticides is of serious concern as it can lead to failure of all efforts geared towards prevention and control of diseases transmitted by *Culex* mosquitoes in North-West Nigeria.

## Introduction

Diseases transmitted by mosquitoes are of major public health concern because they are threats to more than 40% of the world’s population [1]. Despite several years of continuous and appropriate efforts geared towards the control of mosquito-borne diseases (MBDs), they are still responsible for substantial global morbidity and mortality [2,3]. MBDs have kept on extending into geographical regions where they have never existed and re-emerged in areas where control efforts had led to subsidence in previous years [4]. In many regions of the world, the *Anopheles*, *Aedes* and *Culex* genura are of particular interest in public health due to their roles in disease transmission, however, studies have focused more on *Anopheles* and *Aedes* spp. due to their impacts in malaria and arboviral disease transmission. Unfortunately, the *Culex* spp. is the most abundant [5] and most widely distributed mosquito species in the world [6]. The abundance of *Culex* mosquitoes is dictated by their ability to breed in a wide range of water bodies especially heavily polluted water bodies [7].

The *Culex* genus is home to 769 species of mosquitoes [8] known to be vectors of diseases that include Dengue fever and West Nile fever in sub-Saharan Africa [9]. Apart from their roles in transmission of diseases, *Culex* mosquitoes possess high biting ability that has been reported to exceed 10 bites/person/night [10] thereby creating a high level of nuisance and discomfort to humans. Out of all the *Culex* spp., *Cx*. *pipiens* complex predominates in sub-Saharan Africa [6] and *Cx*. *quinquefasciatus* remains the most abundant sibling species of the complex in the tropical and sub-tropical region of the world [11] including sub-Saharan Africa, where it has been implicated to a little extent in the transmission of lymphatic filariasis [12], a disease majorly transmitted by *Anopheles* mosquitoes in sub-Saharan Africa [13].

LF is the second most common cause of permanent disability worldwide [14] and it is majorly transmitted by *Anopheles* mosquitoes in Nigeria with *Culex* mosquitoes contributing to its transmission to an extent [12]. It is a public health scourge that has continued to attract attention from major world organizations due to its impacts in more than 80 countries [15]. Control and elimination of LF transmission have majorly been done through repeated mass drug administration (MDA) of ivermectin or diethylcarbamazine citrate in combination with albendazole [16]. As much as there are positive feedbacks with the use of MDA, total elimination of LF has not been achieved in sub-Saharan Africa. The inability of MDA to achieve total elimination of LF and fear of resurgence in areas that have been declared LF-free in the past has raised concerns about the role of vector control as a complementary tool to MDA in LF elimination [16]. Likewise, Jones *et al*., [1] earlier stated that, “If control and elimination targets for mosquito-borne neglected tropical diseases are to be met over the next decade, vector control interventions need to play an increasingly prominent role”. Complementing MDA with vector control could be very helpful in achieving total elimination in areas where LF is still a challenge, however, vector control is not without its attendant challenges. Topmost on the list of challenges faced in vector control in sub-Saharan Africa is insecticide resistance (IR) in mosquitoes.

Resistance to insecticides in different mosquito species is a huge challenge that has continued to threaten vector control of mosquito-borne diseases [17]. It is a major problem due to the rate at which IR to recommended insecticides develop and spread in all countries in sub-Saharan Africa [18]. Resistance in *Culex* spp. in particular has been reported in several west African countries [19,20]. If IR is not properly managed, it could lead to the failure of vector control efforts in the prevention, control and elimination of mosquito-borne diseases [21]. Likewise, the development and spread of insecticide resistance in *Culex* species could be a huge challenge if *Culex*-borne diseases re-emerge in areas where elimination had previously been achieved or is introduced into communities where MDA has not yet covered. Notably, IR in *Culex* species could also impact malaria elimination programmes if communities perceive reduced efficacy of IRS and ITN/LLINs due to IR in *Culex* spp. and the usage rate of these tools decline [22]. Therefore, it is imminent to monitor the resistance status of *Culex* spp. in regions such as North-West Nigeria where *Culex* spp. are overabundant and has been implicated in the transmission of some diseases. Thus, this study investigated the (1) resistance status of *Cx*. *quinquefasciatus* to pyrethroids (2) pyrethroid resistance mechanisms in *Cx*. *quinquefasciatus* and (3) pyrethroid resistance intensity in *Cx*. *quinquefasciatus* in North-West Nigeria.

## Materials and methods

The study was conducted between December 2020 and April 2021 in Jigawa, North-West Nigeria. This period coincided with the harmattan and dry period in the region. The resistance status of *Culex* populations to permethrin, lambda-cyhalothrin and alphacypermethrin was determined by the WHO resistance bioassay method. The WHO method was also employed for the synergist assay to assess the involvement of Cytochrome-P450 in resistance development in resistant populations. Resistance status was further confirmed with the CDC bottle resistance bioassay (with slight modification) and the same method was used to assess resistance intensity of the populations to the pyrethroids.

### Study area

Jigawa State is one of the poorest states in Nigeria and it is situated in North-West Nigeria; a region in the Sahel savannah ecological zone. It is located at 12.2280° N, 9.5616° E. About 85% of the population live in rural areas while 15% live in towns and cities. One LGA was selected from each of the senatorial zones in the state and the choice of LGA from each zone is determined mainly by lack of data on susceptibility/resistance of *Culex* mosquitoes coupled with relatively high human population of the LGAs. The three (3) Local Government Areas (LGAs) selected for this study; Dutse (11.7024° N, 9.3340° E), Ringim (12.2852° N, 9.4730° E) and Kafin-Hausa LGA (12.1898° N, 9.9242° E) are located in the Central, North-West and North-East parts of the state respectively.

### Mosquito collection and rearing

Considering the fact that the period of collection (December-April) was a season of intense drought in Northern Nigeria, there was difficulty locating larval habitats in Dutse and Ringim but not in Kafin- Hausa. Larval collection was done in different communities within the LGAs and the larval sources were very diverse especially in Kafin-Hausa where there was less difficulty in locating larval habitats. Third and fourth instar larvae of *Cx*. *quinquefasciatus* were collected from gutters and open ditches. The larvae were transported to the insectary at Federal University Dutse, Jigawa State and reared at a temperature of 27°C ± 2°C and relative humidity of 78% ± 3% until they became adults. Emerged adult mosquitoes were collected into adult mosquito cages and fed with 10% sugar solution before exposure. Female samples from the populations were separated from the male samples using morphological features and the females were used for the study.

### WHO insecticide resistance bioassay

Insecticide resistance bioassay was conducted using World Health Organization standard procedures [23]. Insecticide papers treated with permethrin (0.75%), lambda-cyhalothrin (0.05%) and alphacypermethrin (0.05%) were obtained from Vector Control Research Unit, Universiti Sains Malaysia. Non-blood fed female adult *Cx*. *quinquefasciatus* aged 2–5 days were exposed to insecticide treated papers for 60 min at 27 ± 1°C and 80% ± 2% relative humidity. Twenty to twenty-five (20–25) female *Cx*. *quinquefasciatus* were introduced into a holding tube in 4 replicates for 10 mins after which the mosquitoes were gently transferred into experimental tubes and monitored at different time intervals (10, 15, 20, 30, 40, 50, 60 minutes) for the number of mosquitoes “knocked-down”. After one hour of exposure, mosquitoes were transferred into holding tubes and provided with cotton wool saturated with 10% sugar solution. Batches of wild field strains exposed to pyrethroid control papers were used as control. Mortality was recorded after 24 hours and resistance status was determined. Resistant (alive) samples were collected and placed in a freezer under -20°C for 30mins to incapacitate them, after which all samples were stored separately in 1.5ml Eppendorf tubes (Eppendorf International, Germany) containing silica beads.

### WHO synergist bioassay

Synergist assay was conducted in line with WHO protocol [23] to determine the involvement of metabolic enzymes in resistance development. A cohort of *Cx*. *quinquefasciatus* populations already confirmed to be resistant were pre-exposed to 5% piperonyl butoxide (PBO) impregnated papers for 60 mins before they were transferred into exposure tubes containing insecticide impregnated-papers. The procedure for WHO resistance bioassay was then followed as explained earlier.

### CDC insecticide resistance and resistance intensity bioassay

CDC insecticide resistance bioassay was conducted to corroborate the WHO resistance bioassay. The protocol stated in “guideline for evaluating insecticide resistance in vectors using the CDC bottle bioassay” [24] was employed but with slight modification. The CDC bottles were coated with permethrin (21.5μg/bottle), lambda-cyhalothrin (12.5 μg/bottle) and alphacypermethrin (12.5 μg/bottle) and left overnight to dry. Female *Anopheles* mosquitoes were introduced into the coated bottles and the experiment was left for 30 mins. After 30 min of exposure, all mosquitoes were transferred into paper cups and provided 10% sugar solution soaked in cotton wool. The experiment was left for 24 hr before dead and living samples were recorded as susceptible and resistant samples respectively. The experiment was left for 24 hr before recordings of results so as to be comparable with results from the WHO resistance bioassay.

The protocol for CDC resistance intensity assay is the same as described for the CDC bottle resistance bioassay [24] with resistance intensity determined at concentrations of 2x, 5x and 10x of diagnostic concentrations of the three (3) insecticides.

### Morphological and molecular identification of *Cx*. *quinquefasciatus*

Morphological identification of sub-samples of the three populations was done under a dissecting microscope and identified to species level using morphological keys by Rueda [25]. Genomic DNA of individual mosquitoes was extracted as described by Livak [26]. Molecular identification was conducted with Polymerase Chain Reaction (PCR) as described by Smith and Fonseca [27] using three primers; ACEquin (5’CCTTCTTGAATGGCTGTGGCA-3’), ACEpip (5’-GGAAACAACGACGTATGTACT-3’) and B1246s (5’TGGAGCCTCCTCTTCACGG-3’). PCR products were amplified on 1.5% agarose gel and visualized under Ultra Violet light in a gel documentation machine. Also, presence of *Kdr* “*Leu–phe*” mutation was analyzed using the procedure described by Martinez-Torres *et al*., [28]. Hundred (100) resistant samples (40 samples from Kafin-Hausa and 30 samples each from Dutse and Ringim) were selected for *Kdr* analysis.

### Data analysis

For the WHO insecticide resistance bioassay, percentage mortality was calculated and insecticide resistance status was determined using WHO criteria [23]; the population was considered susceptible when mortality ≥ 98%, the population was suspected to be resistant when mortality is ≥ 90% but < 98% and the population was considered resistant when mortality is < 90%. Knockdown time (KDT_50_ and KDT_95_) was determined by log-time-probit model. Also, for the CDC bottle bioassay, percentage mortality was calculated and 98%–100% mortality after 24 hr at the recommended diagnostic dose indicates susceptibility; 80%–97% mortality after 24 hr at the recommended diagnostic dose suggests the possibility of resistance and mortality <80% after 24 hr at the recommended diagnostic dose suggests resistance. Resistance intensity was classified as low, moderate, high and very high when full susceptibility is not achieved at 1x, 2x, 5x and 10x of recommended diagnostic concentrations respectively. In situations where mortality in control was ≥ 5% but < 20%, Abbott’s formula [29] was employed in correction of mortality in the test experiment. T-test was used to determine the significant difference between percentage mortality to the different insecticides. Pearson’s correlation was employed to determine the relationship between percentage mortality recorded in the WHO bioassay method and the CDC bottle bioassay. All data were analyzed using Statistical Package for Social Sciences (IBM SPSS) software version 22.0.

## Results

Morphological identification of cohort from the samples showed that all the *Culex* spp. belonged to *Cx*. *pipiens* complex. Further molecular analysis of 105 samples (35 samples each from Dutse, Ringim and Kafin-Hausa) showed that only *Cx*. *quinquefasciatus* was present in the three LGAs. Also, hundred (100) resistant samples from the three LGAs (more samples were selected from Kafin-Hausa due to the level of resistance) were analyzed for presence of *Kdr* “Leu-Phe” mutation (“*Kdr-w*est”). Out of the 100 resistant samples analyzed, *Kdr*-west mutation was not found in any of the resistant *Cx*. *quinquefasciatus* samples.

### WHO resistance bioassay

Resistance status of *Cx*. *quinquefasciatus* populations from Dutse, Ringim and Kafin-Hausa LGAs exposed to permethrin (0.75%), lambda-cyhalothrin (0.05%) and alphacypermethrin (0.05%) are presented in Table 1. The three populations were all resistant to alphacypermethrin while resistance to permethrin was recorded in Dutse and Kafin-Hausa but not in Ringim. *Culex* population from Dutse was susceptible to lambda-cyhalothrin but resistant in Kafin-Hausa. However, resistance to lambda-cyhalothrin was suspected in *Cx*. *quinquefasciatus* population from Ringim. A comparison of mortality values for the different insecticides showed a significantly high t-value (t = 4.497; df = 2; *p* = 0.046) for the difference between the percentage mortality to lambda-cyhalothrin and alphacypermethrin across the three sites.

**Table 1:** Resistance status of *Cx*. *quinquefasciatus* populations from Jigawa, North-West Nigeria exposed to Pyrethroids.

LGA	Insecticide	Mortality (Number exposed)	% Mortality	Resistance status
**Dutse**	permethrin	10 (82)	12.2	**Resistant**
	lambda-cyhalothrin	82 (83)	98.8	**Susceptible**
	alphacypermethrin	56 (87)	64.37	**Resistant**
**Ringim**	permethrin	100 (100)	100	**Susceptible**
	lambda-cyhalothrin	72 (80)	90	**Resistance suspected**
	alphacypermethrin	16 (85)	18.82	**Resistance**
**Kafin-Hausa**	permethrin	63 (81)	77.78	**Resistant**
	lambda-cyhalothrin	68 (81)	83.95	**Resistant**
	alphacypermethrin	36 (89)	40.45	**Resistant**

Population was considered susceptible when mortality ≥ 98%; Population was suspected to be resistant when mortality is ≥ 90% but < 98%; Population was considered resistant when mortality is < 90%.

Knockdown time of *Cx*. *quinquefasciatus* exposed to permethrin, lambda-cyhalothrin and alphacypermethrin are shown in Table 2. Analysis of knockdown time (KdT) showed that KdT values were lowest to permethrin, lambda-cyhalothrin and alphacypermethrin in Dutse, Ringim and Kafin-Hausa populations respectively.

**Table 2:** KdT_50_ and KdT_95_ values of *Cx*. *quinquefasciatus* populations from Jigawa, North-West Nigeria exposed to Pyrethroids.

LGA	Insecticide	KdT_50_ (CI 95%)	KdT_95_ (CI 95%)
**Dutse**	Permethrin	NA	NA
	Permethrin + PBO	47.79 (45.01–51.07)	81.08 (74.68–89.78)
	Lambda-cyhalothrin	73.09 (66.12–85.24)	108.85 (93.87–140.13)
	Lambda-cyhalothrin + PBO	ND	ND
	Alphacypermethrin	99.74 (80.76–162.14)	147.38 (112.01–266.51)
	Alphacypermethrin + PBO	70.73 (64.47–80.93)	108.7 (94.91–132.4)
**Ringim**	Permethrin	92.05 (78.20–131.27)	139.77 (110.80–229.79)
	Permethrin + PBO	ND	ND
	Lambda-cyhalothrin	81.32 (69.07–168.36)	117.54 (90.02–321.89)
	Lambda-cyhalothrin + PBO	93.50 (78.42–129.03)	145.11 (115.25–217.38)
	Alphacypermethrin	304.21 (NA)	516.82 (NA)
	Alphacypermethrin + PBO	83.25 (72.7–103.9)	129.13 (107.33–173.3)
**Kafin-Hausa**	Permethrin	113.41 (NA)	154.66 (NA)
	Permethrin + PBO	60.80 (57.49–65.37)	87.66 (80.22–98.99)
	Lambda-cyhalothrin	146.15 (100.72–399.27)	257.25 (166.55–769.26)
	Lambda-cyhalothrin + PBO	91.51 (73.82–133.59)	188.52 (142.65–301.35)
	Alphacypermethrin	89.57 (76.55–117.94)	137.62 (111.64–195.89)
	Alphacypermethrin + PBO	74.72 (66.77–88.07)	121.59 (103.98–152.55)

CI = Confidence interval for the values; NA = Not available (Indicating where KdT or CI cannot be computed); ND = Not done (indicating instance where synergist assay was not done due to record of susceptibility when population was exposed to insecticide only).

### Synergist assay

**Fig 1:**
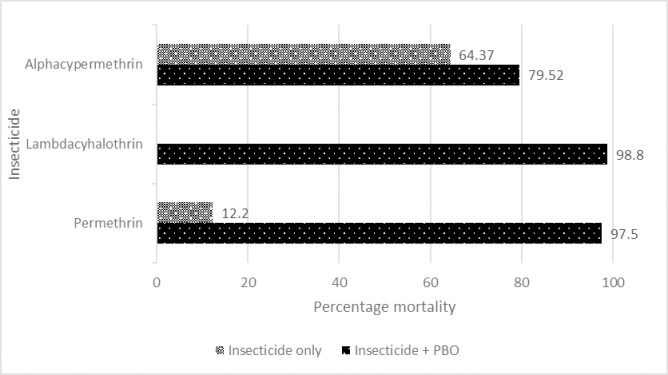
Percentage mortality of *Culex quinquefasciatus* population from Dutse LGA exposed to PBO and selected pyrethroids.

**Fig 2:**
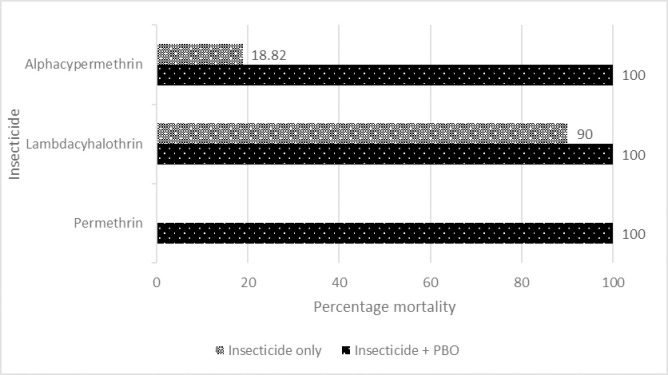
Percentage mortality of *Culex quinquefasciatus* population from Ringim LGA exposed to PBO and selected pyrethroids.

**Fig 3:**
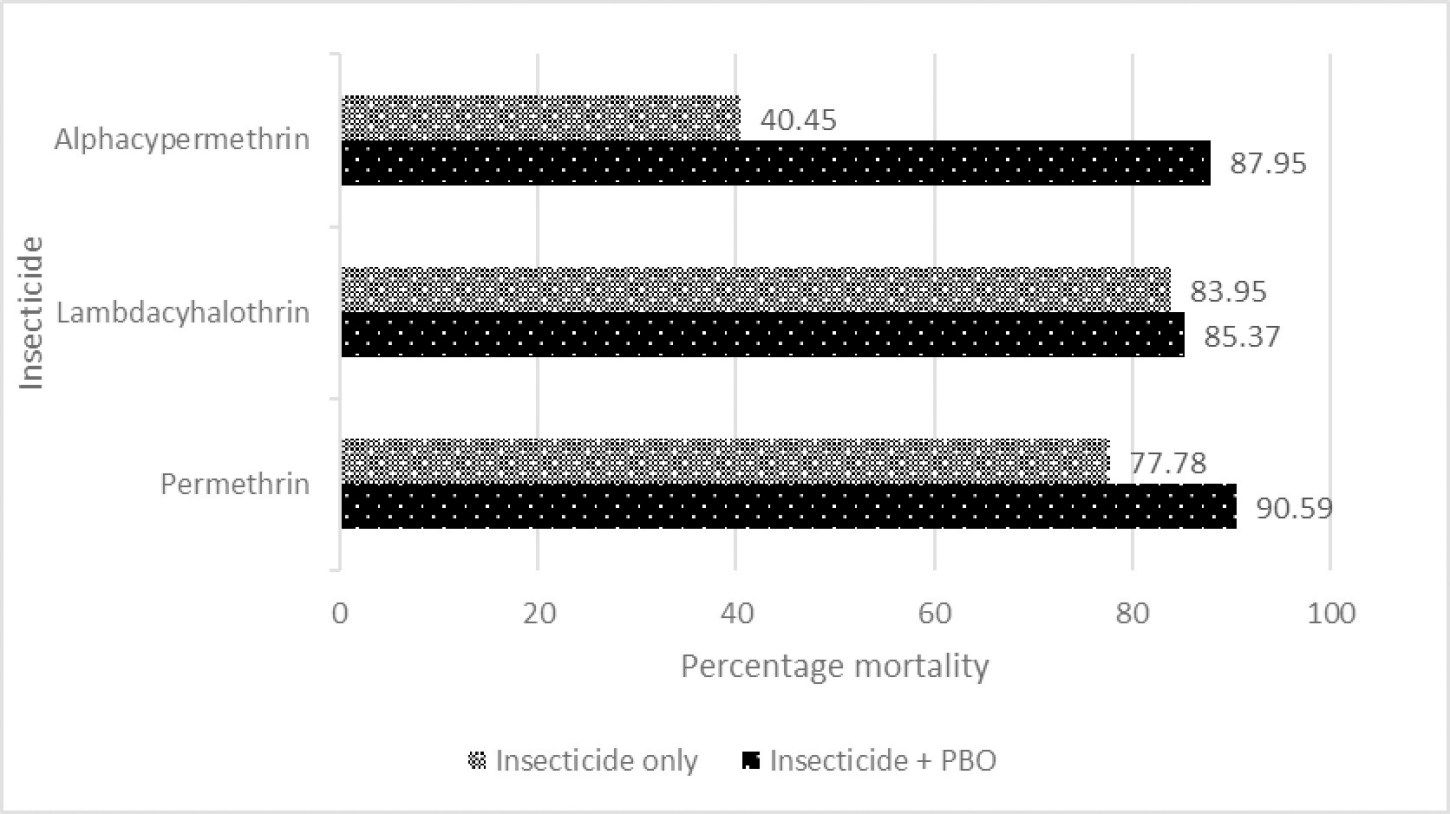
Percentage mortality of Culex quinquefasciatus population from Kafin-Hausa LGA exposed to PBO and selected pyrethroids.

Cohorts of resistant populations were pre-exposed to PBO before exposure to insecticides. In Dutse, there was a remarkable increase in mortality (from 12.2% to 97.5%) when the population was exposed to permethrin after pre-exposure to PBO (Fig 1). Pre-exposure of Ringim *Cx*. *quinquefasciatus* population led to total susceptibility to all insecticides (Fig 2) while increased mortality to permethrin (77.78% to 90.59%), lambda-cyhalothrin (83.95% to 85.37%) and alphacypermethrin (40.45% to 87.95%) were also observed in Kafin-Hausa (Fig 3).

### CDC resistance intensity bioassay

Resistance studies using CDC resistance bioassay corroborated results from WHO resistance bioassay with resistance recorded in all study sites. There was a significantly high positive correlation (R^2^ = 0.728, *p* = 0.026) between the result of percentage mortality after 24 hr in the WHO resistance bioassay and the CDC bottle bioassay. The level of resistance was also generally higher in Kafin-Hausa to the 3 pyrethroids and lowest in Ringim in a similar pattern to results obtained in WHO resistance bioassay. Likewise, resistance to alphacypermethrin was more pronounced than resistance to permethrin and lambda-cyhalothrin across all sites. The intensity of resistance to the three (3) insecticides in resistant populations of *Cx*. *quinquefasciatus* is presented in Tables 3–5. Resistance intensity to permethrin was low at Ringim with percentage mortality (96%) close to full susceptibility at 1x of diagnostic concentration while moderate resistance intensity was recorded in both Dutse and Kafin-Hausa with full susceptibility achieved at 5x of diagnostic concentration (Table 3). Contrary to the result obtained for permethrin, resistance intensity to lambda-cyhalothrin was very high in Kafin-Hausa (87.5%) with full susceptibility not achieved even at 10x of diagnostic concentration, however, intensity in Ringim was moderate with 100% mortality at 5x (Table 4). Also, resistance intensity to alphacypermethrin was high in Kafin-Hausa but moderate in both Dutse and Ringim (Table 5).

**Table 3:** Resistance intensity status of *Culex quinquefasciatus* after 24-hr post exposure to 1x, 2x, 5x and 10x of diagnostic concentration of permethrin in CDC bottle bioassay.

Location	Permethrin concentration					
		1x (21.5μg)	2x (43μg)	5x (107.5μg)	10x (215μg)	Intensity status
Dutse	No. exposed	80	80	80	80	Moderate
	24h %Mortality	66.25	85	100	ND	
Ringim	No. exposed	80	80	80	80	Low
	24h %Mortality	96	100	ND	ND	
Kafin-Hausa	No. exposed	80	80	80	80	Moderate
	24h %Mortality	55	76.25	99	ND	

98%–100% mortality indicates susceptibility; 80%–97% mortality suggests the possibility of resistance; mortality <80% suggests resistance. ND = Not done (This applies to cases where susceptibility has been achieved at a lower concentration). Intensity status is low when susceptibility is not achieved at 1x of diagnostic dose; moderate when susceptibility is not achieved at 2x of diagnostic dose; high when susceptibility is not achieved at 5x of diagnostic dose; very high when susceptibility is not achieved at 10x of diagnostic dose.

**Table 4:** Resistance intensity status of *Culex quinquefasciatus* after 24-hr post exposure to 1x, 2x, 5x and 10x of diagnostic concentration of lambda-cyhalothrin in CDC bottle bioassay.

Location	lambda-cyhalothrin concentration					
		1x (12.5μg)	2x (25 μg)	5x (62.5 μg)	10x (125 μg)	Intensity status
Dutse	No. exposed	80	80	80	80	Low
	24h %Mortality	80	100	ND	ND	
Ringim	No. exposed	80	80	80	80	Moderate
	24h %Mortality	82.5	92	100	ND	
Kafin-Hausa	No. exposed	80	80	80	80	Very high
	24h %Mortality	75	80	82	87.5	

98%–100% mortality indicates susceptibility; 80%–97% mortality suggests the possibility of resistance; mortality <80% suggests resistance. ND = Not done (This applies to cases where susceptibility has been achieved at a lower concentration). Intensity status is low when susceptibility is not achieved at 1x of diagnostic dose; moderate when susceptibility is not achieved at 2x of diagnostic dose; high when susceptibility is not achieved at 5x of diagnostic dose; very high when susceptibility is not achieved at 10x of diagnostic dose.

**Table 5:** Resistance intensity status of *Culex quinquefasciatus* after 24-hr post exposure to 1x, 2x, 5x and 10x of diagnostic concentration of alphacypermethrin in CDC bottle bioassay.

Location	alphacypermethrin concentration					
		1x (12.5μg)	2x (25 μg)	5x (62.5 μg)	10x (125 μg)	Intensity status
Dutse	No. exposed	80	80	80	80	Moderate
	24h %Mortality	42.5	60	100	ND	
Ringim	No. exposed	80	80	80	80	Moderate
	24h %Mortality	10	67.5	100	ND	
Kafin-Hausa	No. exposed	80	80	80	80	High
	24h %Mortality	30	60	82.5	100	

98%–100% mortality indicates susceptibility; 80%–97% mortality suggests the possibility of resistance; mortality <80% suggests resistance. ND = Not done (This applies to cases where susceptibility has been achieved at a lower concentration). Intensity status is low when susceptibility is not achieved at 1x of diagnostic dose; moderate when susceptibility is not achieved at 2x of diagnostic dose; high when susceptibility is not achieved at 5x of diagnostic dose; very high when susceptibility is not achieved at 10x of diagnostic dose.

## Discussion

Monitoring resistance status of *Cx*. *quinquefasciatus* is not only essential to vector control of diseases transmitted by *Culex* mosquitoes but highly important in the prevention and control of all mosquito-borne diseases. This is due to the high nuisance value and biting ability of *Culex* mosquitoes which generally impact negatively on community’s perception to vector control strategies aimed at control of diseases transmitted by other mosquito species. Proper management of these challenges posed by *Culex* mosquitoes can only be done through a sound assessment of the current status of resistance and resistance intensity of *Culex* mosquitoes to recommended insecticides, thus, this study assessed the resistance status of *Cx*. *quinquefasciatus* in Jigawa, North-West Nigeria to permethrin, lambda-cyhalothrin and alphacypermethrin.

Molecular analysis of mosquito samples showed that *Culex* spp. collected from the three study locations were all *Cx*. *quinquefasciatus*. Other sibling species of *Cx*. *pipiens* complex were not recorded in the present study. While *Cx*. *pipiens* has been recorded in the work of Rabi’u and Ahmed [30] in the Northern region of Nigeria, prevalence of *Cx*. *quinquefasciatus* is well reported in several other studies in Nigeria [22,31].

Resistance of *Cx*. *quinquefasciatus* was recorded in all three locations with populations from Dutse and Kafin-Hausa resistant to permethrin. Alphacypermethrin resistance was recorded in all populations while resistance to lambda-cyhalothrin was confirmed only in Kafin-Hausa. Resistance of *Cx*. *quinquefasciatus* to permethrin [31], lambda-cyhalothrin [32] and other pyrethroids [33,34] have been reported in several locations in Nigeria. Abba *et al*., [35] had earlier reported “suspected resistance” of *Culex* mosquitoes to alphacypermethrin in locations close to the present study site, however, this is the first report confirming resistance of *Cx*. *quinquefasciatus* to alphacypermethrin in Nigeria. Report of resistance in *Cx*. *quinquefasciatus* to alphacypermethrin is a major development because alphacypermethrin is the pyrethroid insecticide employed in the treatment of new nets proposed for the country in 2022 [22]. Similarly, resistance to permethrin may be easily attributed to an increase in the selection pressure due to increase in volume of permethrin in the environment occasioned by current use of permethrin-treated nets in the country. More attention should be paid to assessing the level of resistance of different species of mosquitoes especially *Culex* species to alphacypermethrin, considering the fact that resistance in *Culex* spp. may impact negatively on human perception as regards efficacy of treated nets aimed at control of other diseases.

Considering the spread of resistance in the three populations, resistance mechanisms involving *Kdr* “Leu-Phe” mutation (*Kdr* west) and impact of cytochrome P450 (by pre-exposure to PBO) were analyzed. “*Kdr*-west” mutation was not found in any of the resistant *Cx*. *quinquefasciatus* samples. Absence of “*Kdr*-west” in *Cx*. *quinquefasciatus* from Nigeria had earlier been reported in the work of Ojianwuna *et al*., [22]. This suggests the possible involvement of other resistance mechanisms in resistance development in the *Cx*. *quinquefasciatus* populations. Exposure of the *Cx*. *quinquefasciatus* populations to PBO before further exposure to the pyrethroids led to increased mortality in all populations. Earlier studies had reported similar results [1,36] and this suggests the involvement of cytochrome P450 in resistance development in the *Cx*. *quinquefasciatus* populations. Cytochrome P450 is one of the metabolic enzymes that sequesters pyrethroids thereby assisting vectors to evade harmful impacts of the insecticides [37,38]. Pre-exposure of vector populations to PBO leads to reduction in the impact or volume of cytochrome P450 thereby allowing for assessing its involvement in resistance development [15]. Its involvement in resistance development in *Cx*. *quinquefasciatus* as recorded in this study has also been documented in other regions of the world [39,40,41]. The implication of cytochrome P450 in resistance development supports the use of PBO in combination with different pyrethroids for control of mosquitoes.

Using the CDC bottle assay, resistance to permethrin, lambda-cyhalothrin and alphacypermethrin was further confirmed except in Ringim population when exposed to permethrin. While monitoring the development and spread of resistance in vectors is essential, monitoring the intensity of resistance in resistant populations of vectors is of paramount importance. Resistance intensity provides information on the strength of resistance in the vector population, thereby providing relevant information needed for management of insecticide resistance. Result of resistance intensity using CDC bottle bioassay in this present study showed that resistance intensity of *Cx*. *quinquefasciatus* in Kafin-Hausa was very high with resistance still recorded at 10x of the diagnostic dose of lambda-cyhalothrin. High resistance intensity in Kafin-Hausa to two of the pyrethroids tested calls for serious attention. It is indicative of the limitations of pyrethroid-based tools in vector control of mosquitoes in Kafin-Hausa and North-West Nigeria at large. This supports the clamour for other vector control tools which are not insecticide-based; especially larval source management through habitat modifications.

In as much as the synergist assay implicates cytochrome P450 in resistance development, further analysis of the level of metabolic enzymes through enzymes assays would have been interesting, however, this was not done and it highlights one of the limitations of this study. Another limitation of the present study is inability to characterize the second type of knockdown mutation known as ‘*Kdr-East*”. *Kdr-East* is a mutation arising from the substitution of leucine to serine on the 1014 gene [37]. It a mutation known to be prevalent in Eastern Africa but recent researches have found the presence of the mutation in some mosquito populations in West Africa albeit in very low frequency.

Fear of re-emergence of *Culex*-borne diseases coupled with high nuisance level of *Culex* mosquitoes dictated by their persistent biting ability have made concerns about *Culex* mosquitoes in sub-Saharan Africa a genuine concern [42]. Furthermore, the intensity of resistance in *Cx*. *quinquefasciatus* to pyrethroids in this study calls for serious introspection into alternatives to pyrethroids in the control of *Cx*. *quinquefasciatus* in North-West Nigeria.

## Conclusion

*Cx*. *quinquefasciatus* is the dominant *Culex* species in Dutse, Ringim and Kafin-Hausa LGA in North-West Nigeria. There is widespread insecticide resistance (using WHO resistance method) in *Cx*. *quinquefasciatus* populations from Jigawa state, North-West Nigeria to pyrethroids; permethrin, lambda-cyhalothrin and alphacypermethrin. Resistance studies using CDC bottle bioassay further confirmed resistance in the *Cx*. *quinquefasciatus* populations. *Kdr*-west mutation was not detected in any of the sampled populations. Pre-exposure to PBO implicated cytochrome P450 in development of resistance in the three populations. Also, there was very high resistance intensity to lambda-cyhalothrin in Kafin-Hausa. Control of *Cx*. *quinquefasciatus* populations from this region of Nigeria can be addressed with combination of pyrethroids and PBO, however, the development, spread and intensity of insecticide resistance in *Cx*. *quinquefasciatus* calls for effective integrated management approach in the control of mosquitoes in Jigawa, North-West Nigeria. More importantly, proper integration of non-insecticide-based methods should be looked into.

## Supporting information

S1 Data Result of insecticide resistance/susceptibility test to permethrin, lambda-cyhalothrin and alphacypermethrin using CDC bottle bioassay at diagnostic concentrations. (XLSX)

S2 Data Result of insecticide resistance/susceptibility test to permethrin, lambda-cyhalothrin and alphacypermethrin using CDC bottle bioassay at X2 of diagnostic concentrations. (XLSX)

S3 Data Result of insecticide resistance/susceptibility test to permethrin, lambda-cyhalothrin and alphacypermethrin using CDC bottle bioassay at X5 of diagnostic concentrations. (XLSX)

S4 Data Result of insecticide resistance/susceptibility test to permethrin, lambda-cyhalothrin and alphacypermethrin using CDC bottle bioassay at X10 of diagnostic concentrations. (XLSX)

S5 Data Result of insecticide resistance/susceptibility test to permethrin, lambda-cyhalothrin and alphacypermethrin using WHO susceptibility bioassay at diagnostic concentrations. (XLSX)

S6 Data Result of synergist assay using WHO susceptibility bioassay method. (XLSX)
